# Barriers and facilitators for reducing low‐value home‐based nursing care: A qualitative exploratory study among homecare professionals

**DOI:** 10.1111/jan.16381

**Published:** 2024-08-22

**Authors:** Milou Cremers, Benjamin Wendt, Getty Huisman‐de Waal, Leti van Bodegom‐Vos, Simone A. van Dulmen, Elise Schipper, Monique van Dijk, Erwin Ista

**Affiliations:** ^1^ Department of Internal Medicine, Section of Nursing Science, Erasmus MC University Medical Center Rotterdam Rotterdam the Netherlands; ^2^ Radboud Institute for Health Sciences, IQ Healthcare Radboud University Medical Center Nijmegen The Netherlands; ^3^ Department of Biomedical Data Sciences Leiden University Medical Center Leiden The Netherlands; ^4^ Bravis Hospital Bergen op Zoom The Netherlands; ^5^ Division of Pediatric Intensive Care, Department of Neonatal and Pediatric Intensive Care, Erasmus MC‐Sophia Children's Hospital University Medical Center Rotterdam Rotterdam The Netherlands

**Keywords:** appropriate care, barriers and facilitators, de‐implementation, homecare, low‐value care, nurse, tailoring strategies

## Abstract

**Aim:**

To explore barriers and facilitators for reducing low‐value home‐based nursing care.

**Design:**

Qualitative exploratory study.

**Method:**

Seven focus group interviews and two individual interviews were conducted with homecare professionals, managers and quality improvement staff members within seven homecare organizations. Data were deductively analysed using the Tailored Implementation for Chronic Diseases checklist.

**Results:**

Barriers perceived by homecare professionals included lack of knowledge and skills, such as using care aids, interactions between healthcare professionals and general practitioners creating expectations among clients. Facilitators perceived included reflecting on provided care together with colleagues, clearly communicating agreements and expectations towards clients. Additionally, clients' and relatives' behaviour could potentially hinder reduction. In contrast, clients' motivation to be independent and involving relatives can promote reduction. Lastly, non‐reimbursement and additional costs of care aids were perceived as barriers. Support from organization and management for the reduction of care was considered as facilitator.

**Conclusion:**

Understanding barriers and facilitators experienced by homecare professionals in reducing low‐value home‐based nursing care is crucial. Enhancing knowledge and skills, fostering cross‐professional collaboration, involving relatives and motivating clients' self‐care can facilitate reduction of low‐value home‐based nursing care.

Implications for profession and patient care: De‐implementing low‐value home‐based nursing care offers opportunities for more appropriate care and inclusion of clients on waitlists.

**Impact:**

Addressing barriers with tailored strategies can successfully de‐implement low‐value home‐based nursing care.

**Reporting Method:**

The Consolidated Criteria for Reporting Qualitative Research checklist was used.

No patient or public contribution.

**Contribution of the paper**

- First insights into low‐value home‐based nursing care from the perspectives of homecare professionals.
- In‐depth insights into barriers and facilitators contributing to the provision of low‐value home‐based nursing care.
- These insights can serve as a foundation for developing tailored de‐implementation strategies in home‐based nursing care.

## 1 INTRODUCTION

Demand for home‐based nursing care is rising, driven by the global ageing population and an increase in multi‐morbidity (Rudnicka et al., 2020). Concurrently, there is shift in nursing care from hospitals to the home environment, and older people tend to stay at home longer, often requiring more complex care (van de Maat et al., 2015). Alongside the increased demand, there is a global shortage of nurses and nursing assistants, which is expected to worsen in the near future. This shortage is also evident in the homecare setting in the Netherlands, where a shortage of 11% (10,600) of nurses and nursing assistants is anticipated by 2027 (Grijpstra et al., 2020). At the same time, some care provided by homecare nurses and nursing assistants is not evidence‐based or not beneficial for the client. Despite the absence of an international consensus on the precise definition of what exactly constitutes ‘low‐value care’, the available literature identifies three categories of care: (1) ineffective care, which causes more harm than good; (2) inefficient care, which is not as effective as it could be, is continued for an extensive period, is administered too frequently or could be replaced by a care aid and (3) unwanted care, which does not improve clients' conditions or align with their preferences (Verkerk, Tanke, et al., 2018). An example of low‐value care in nursing, in general, is ‘dressing a primarily closed wound with bandages’, when a closed wound does not need bandaging (Osorio et al., 2019). It is a waste of resources and time that could be better spent on providing more evidence‐based care (Berwick & Hackbarth, 2012). To address future challenges in homecare, it is important to reduce low‐value home‐based nursing care practices.

A recent survey study among homecare professionals in the Netherlands found that the majority (59%) experienced low‐value home‐based nursing care on a daily basis (Wendt et al., 2023). Some low‐value practices occur regularly in homecare settings, such as putting on compression stockings when a client can do it with the use of a care aid or washing clients with water and soap by default (Verkerk, Huisman‐de Waal, et al., 2018; Wendt et al., 2023). Reducing low‐value care in home‐based nursing care can create opportunities to provide more appropriate care, based on increased clinical expertise, evidence‐based practice, context‐specificity and person‐centred care (Robertson‐Preidler et al., 2017).

## 2 BACKGROUND

In the Netherlands, home‐based nursing care is provided to clients with chronic illnesses, dementia, those in the final stages of palliative care and individuals requiring temporary care after hospital discharge (Centre for Public Impact, 2018). All residents and non‐residents paying Dutch income tax must purchase health insurance from a private insurer. However, certain care services, such as care aids, may necessitate an additional contribution from residents (Wammes et al., 2020). In the Netherlands, homecare professionals collaborate closely with clients, families and other disciplines such as social workers and general practitioners (GPs) (Centre for Public Impact, 2018; Rosendal et al., 2019). GPs play a central role in primary care as gatekeepers who refer clients to specialist hospital care or home‐healthcare when necessary. They typically operate independently or in self‐employed partnerships (Wammes et al., 2020). Such collaborative efforts between homecare professionals, clients, families and other disciplines, including GPs, could effectively address the reduction of low‐value home‐based nursing care.

The process of reducing low‐value care is known as de‐implementation. Two types of de‐implementation can be distinguished: (1) the current practice can be substituted with an alternative practice, for example by implementing a care aid for putting on compression stockings and de‐implementing providing the care by homecare professionals and (2) new knowledge recommends to eliminate the current provided practice, for example when the care is not of benefit to the client and does not justify the cost (van Bodegom‐Vos et al., 2017). However, changing or abandoning current clinical practices is often more challenging than adopting new ones. This applies universally to behaviour modification, even when evidence has demonstrated that current practices hold little or no value (van Bodegom‐Vos et al., 2017; van Dulmen et al., 2020). De‐implementing requires a structured plan encompassing multiple strategies, along with a comprehensive understanding of possible barriers and facilitators across all involved stakeholders (Augustsson et al., 2021; van Bodegom‐Vos et al., 2017; van Dulmen et al., 2020). It should be noted, though, that the value of care can vary depending on the client and the client's specific situation (Ingvarsson et al., 2022). To develop tailored de‐implementation strategies to reduce low‐value home‐based nursing care, knowledge about barriers and facilitators in homecare is needed.

Previous understanding of barriers for nurses included attitudes such as reluctance to use recommended guidelines due to time restraints and tendency to prioritize client expectations and preferences, along with having to deal with the impact of nursing culture and work routines (Halm, 2022; van Achterberg et al., 2008). Examples of facilitators include providing education on low‐value care practices, nurturing the desire to learn and restrict low‐value care practices, and fostering a positive environment for communication and collaboration between healthcare professionals (Halm, 2022; van Dulmen et al., 2020). Specific knowledge about barriers and facilitators in the context of home‐based nursing care is currently lacking, and acquiring this knowledge is imperative to effectively de‐implement low‐value home‐based nursing care practices.

A survey study among Dutch homecare professionals revealed influencing factors for providing low‐value home‐based nursing care, including instances where low‐value care is ‘prescribed by a general practitioner’, documented in the client's care plan and consequently carried out, as well as situations where homecare professionals aim to offer something to the client (Wendt et al., 2023). However, as indicated by Wendt et al. (2023), a more in‐depth insight of these influencing factors is necessary to specifically and qualitatively explore the perceived barriers and facilitators influencing the provision of low‐value home‐based nursing care. This insight is crucial for informing strategies aimed at de‐implementing these care practices.

## 3 THE STUDY

This study aims to explore influencing factors—barriers and facilitators—perceived by professionals working in home‐based nursing care for reducing low‐value home‐based nursing care practices.

## 4 METHODS

### 4.1 Design

We conducted a qualitative exploratory study using focus group interviews with homecare professionals and two additional individual interviews with quality improvement staff members. The qualitative design allowed us to obtain detailed and rich data. This study was embedded in the DIMPLE project (De‐IMPLEmentation of low‐value care in home care nursing) and RENEW project (more appropriate care in home‐based nursing care).

### 4.2 Theoretical framework

An interview guide was developed based on the Tailored Implementation for Chronic Diseases (TICD) checklist, which includes the following domains: (1) guidelines, (2) individual health professional factors, (3) professional interaction factors, (4) patient factors, (5) organizational factors, (6) social, political and legal factors and (7) incentives and resources. The TICD checklist serves as a general framework for broad use in different contexts for identifying barriers and facilitators that are crucial for implementing guideline recommendations, such as those for low‐value home‐based nursing care practices (Flottorp et al., 2013). The interview guide can be found in Appendix S1.

### 4.3 Setting, population and recruitment

In total, 27 teams were included, representing seven different homecare organizations in the western (*n* = 1), mid‐central (*n* = 2) and eastern (*n* = 4) regions of the Netherlands. The study population comprised nursing assistants (levels 2 and 3), registered nurses (levels 4 and 6), nurse students, healthcare managers, quality care nurses and quality improvement staff members. These homecare organizations were active in rural and urban settings throughout the Netherlands, had between 8 and 1000 teams and between 95 and 14,000 employees per organization. A homecare team includes 10 to 20 homecare professionals. Inclusion involved both urban and rural teams, larger and smaller healthcare organizations and being active in different regions.

The included teams vary in the level of training, and the care performed by healthcare professionals is adapted to their knowledge and skills. Some care requires nursing expertise, while procedures such as dressing and bathing can be performed by nursing assistants with lower education levels. Nursing assistants focus primarily on daily activities and low complex nursing care. Registered nurses focus on more complex nursing care and coordination of care. In addition, level 6 registered nurse's responsibilities also included conducting needs assessment. This entails evaluating with clients and their networks about what nursing care is needed, aiming to strengthen the ability to care for oneself and to promote, achieve and maintain the performance of necessary activities, known as self‐care (Richard & Shea, 2011; Schwenke, van Dorst, et al., 2023). Appendix S2 provides detailed information on the professions, educational levels and job descriptions of homecare professionals in the Netherlands.

Key individuals—either registered nurses or nurse assistants—within the participating teams were invited by e‐mail. In the e‐mail, we instructed these key individuals to invite one or two colleagues, ensuring variation in education level, years of experience and involvement in low‐value home‐based nursing care practices. This approach aimed to create heterogenic groups. In addition, at least one manager or quality improvement staff member per organization was invited. For each focus group interview, at least 8 to 10 participants were invited, including a manager or a quality improvement staff member. Self‐employed healthcare professionals were excluded because they work only occasionally with the teams and do not play an active role in initiating care or in documenting and adjusting care plans. In cases where a quality improvement staff member could not participate in the focus group interview, they were interviewed individually, as they constituted the minority of the participants and could not be replaced by others. Participation of quality improvement staff members was particularly valuable for providing insights on quality improvement, organizational factors and external factors.

### 4.4 Data collection

Focus group interviews and individual interviews were conducted in person or through video calls when COVID‐19 restrictions were in place between March and June 2022. Each focus group interview was led by one moderator (MC or BW), who guided the discussion, asked probing questions to aid the discussion and requested additional explanations when necessary. A second researcher (ES, ABJ, GHW, SvD) made observational notes on behaviours and interactions to facilitate evaluation and analysis, took notes on the content of the focus group interview and sought clarification based on the notes. Two researchers (MC and ES) independently conducted an individual interview with a quality improvement staff member.

The focus group interviews and individual interviews began with an introduction to the subject. Next, a top ten low‐value home‐based nursing care practices were presented, which were compiled from previous research (Wendt et al., 2023). Appendix S3 presents a list of low‐value home‐based nursing care practices, which varied depending on the organization. Participants were asked whether they performed these practices, what their initial reaction was to the idea of these practices being considered as low value, which motivations there were to continue providing these practices and which factors could help reduce them. The interview guide was employed to pose questions aimed at identifying barriers and facilitators for reducing low‐value home‐based nursing care. All interviews were audio‐recorded and transcribed verbatim.

### 4.5 Data analysis

We employed a directed content analysis with multiple analytical steps (Kibiswa, 2019). Prior to the analysis, a codebook was developed, structured around themes and subthemes derived from the domains of the TICD checklist (Flottorp et al., 2013). Guided by the codebook, we approached the data deductively and clustered insights on barriers and facilitators for low‐value home‐based nursing care (Kibiswa, 2019).

The analysis proceeded through several steps:

1. Two groups of researchers (MC and ES for focus group interviews 1, 2 and 3 and individual interviews 1 and 2; BW and ABJ for focus group interviews 4, 5, 6 and 7) read the transcripts of the interviews.
2. In each group, both researchers independently reviewed the transcripts and extracted relevant segments pertaining to the study. These segments were then organized in alignment with the predetermined themes and subthemes based on the TICD checklist (Flottorp et al., 2013).
3. All extracted content was compared and discussed by MC and BW until a consensus was reached. If consensus could not be reached, a third researcher (EI or GHW) was consulted for resolution. In the end, all relevant domains were comprehensively covered.

Data analysis started after the first focus group interview. The interview guide was reviewed after each focus group interview and individual interview, with a shift of focus to themes that required further examination. This iterative approach was employed to achieve data saturation. During the analysis, we incorporated the ‘guideline factors’ domain in the ‘individual health professional factors’ domain because the data primarily reflected the participants' perspectives on guidelines rather than their reliability, quality or strength. Furthermore, within the ‘patient factors’ domain, we made a distinction between clients, relatives and/or caregivers, recognizing that these parties might differ in their attitudes, behaviours and knowledge. All coding was performed using ATLAS.ti Windows (Version 22.0.11.0).

### 4.6 Ethical considerations

The Medical Ethics Committee of the Erasmus University Medical Center and the Research Ethics committee of the Radboud University Medical Center concluded that ethical approval was not required under Dutch law (MEC‐2021‐0948 and CMO no. 2022‐13545). Prior to inclusion in the study, all participants signed an informed consent form, acknowledging that they had been informed about the purpose of the study, that participation was voluntary and that they could withdraw from the study at any time without providing a reason. They also gave permission to use the collected data and were informed that their data would be used anonymously and confidentially and that it would not be possible to trace the data back to them.

### 4.7 Rigour and reflexivity

To ensure credibility, researcher's triangulation was applied as described in the data analysis. In addition, all participants received a summary of the interviews. They were given the opportunity to respond to the summary, give comments, feedback or make additions to the data. Transferability in a similar setting is considered possible because of a comprehensive description of the characteristics, context of homecare environment and extensive description of data collection. To ensure explicit and comprehensive reporting of the data, we employed the Consolidated Criteria for Reporting Qualitative Research (COREQ) checklist (Tong et al., 2007).

## 5 RESULTS

Seven focus group interviews and two individual interviews were conducted with durations from 74 to 121 min. Three focus group interviews and two individual interviews were conducted online due to COVID‐19 restrictions. In each focus group interview, 6 to 10 participants attended. A summary of the participants’ characteristics is presented in Table 1.

**TABLE 1:** Characteristics of the participants (*n* = 55).

Characteristics	*n*	%
Sex		
Female	48	87.3%
Male	7	12.7%
Age		
<21 years	1	1.8%
21–30 years	20	36.4%
31–40 years	10	18.2%
41–50 years	9	16.4%
>51 years	15	27.2%
Profession		
Nurse student	2	3.6%
Health and welfare assistant (Level 2)	2	3.6%
Certified nursing assistant (Level 3)	11	20%
Registered nurse (Level 4)^a^	13	23.6%
Registered nurse (Level 6)^a^	21	38.2%
Quality improvement staff member	4	7.4%
Healthcare manager	2	3.6%
Experience in home‐based nursing care (years)		
<5 years	18	32.7%
5–10 years	16	29.1%
11–20 years	12	21.8%
>21 years	9	16.4%

^a^ Level 4 is vocationally trained registered nurses, and level 6 is bachelor trained registered nurses. Level 6 has expanded tasks compared to level 4; these can be found in Appendix S2.

### 5.1 Characteristics of the participants

In total, 55 homecare professionals participated in the study. The majority of participants were female (87.3%) and within the age range of 21–30 years (36.4%). The largest proportion were employed as registered nurses at level 4 (23.6%) and level 6 (38.2%) or as certified nursing assistants at level 3 (20%). Most of the participants had less than 5 years (32.7%) or 5 to 10 years (29.1%) of work experience in home‐based nursing care.

### 5.2 Barriers and facilitators

The majority of barriers and facilitators were identified within the domains of ‘individual health professional factors’ and ‘professional interaction factors’. A smaller majority was identified in the domains of ‘client and relatives’ factors’ and ‘external factors’. External factors include the domains of ‘incentives and resources factors’, ‘capacity of organizational changes factors’ and social, political and legal factors’. Table 2 provides an overview of barriers and facilitators identified per domain. Results of barriers and facilitators are presented in order of most found by domain.

**TABLE 2:** Overview of barriers and facilitators for reducing low‐value home‐based nursing care.

TICD^a^	Theme	Homecare professionals experience this as a facilitator…	Homecare professionals experience this as a barrier…
1	Awareness, behaviour and attitude of the individual homecare professional	Setting, defining and communicating goals and boundaries: When you state boundaries and state goals than clients go along Reflecting with you team on (your own) actions/performance Involving of network with de‐implementation process of low‐value care Taking self‐care of the client in to account (when conducting needs assessment) It is motivating seeing the client's benefits Being aware of ‘low‐value home‐based nursing care’	Working on a routine basis, without reflecting on your own (and team's) actions Not using a method to set, measure and/or evaluate health outcomes or goals (e.g. RUMBA^b^) Having difficulty going against clients, carers and/or family members Wanting to offer clients, carers and/or family members ‘something’. Wanting to take care of clients Differing or conflicting views on care Care is provided faster when a homecare professional takes over Fear of losing work: for example the loss of hours and/or payment Peer pressure Experience the recommended care is no more efficient than the current provided care To follow guidelines, the benefits must be significant in comparison to the disadvantages Retaining client in care longer for social monitoring Aware that care is low‐value, but still perform it, for example because general practitioner prescribe it
	Guidelines, knowledge, skills and variation in care of the individual homecare professional	Getting information about guideline updates in general Highlighting adjustments in the recommendation: for example mark changes in red in the guideline in general Being up‐to‐date of care innovations/technology/aids The profession of homecare professionals is evolving, with professionals becoming more knowledgeable Providing courses or training to develop skills Shadowing colleagues to peer and learn from another Receiving guidance in conducting needs assessments Managing expectations (of clients, family and/or caretakers)	Lack of clarity of the guideline, such as lack of a summary and specification: for example information about how often and when an intervention should take place Lack of applicability of the recommended care, such as that the recommend care is not practical in the home‐based context Lack of (digital) skills Lack of sense of urgency to change Lack of professional autonomy Falling back in old patterns if new practices are not yet embedded and not repeated regularly Variation in needs assessments
2	Clients' behaviour and attitude	Clients who want to be independent	Hospitalization of clients (e.g. more passive attitude, receiving long‐term care, want to retain habits) Clients are more empowered and outspoken Clients feeling they have the right to receive care Clients switching homecare organization when reducing care Clients gradually demand more care (accept little at first and then try to expand or reversing previous agreements) Clients using nursing care as social contact Clients appreciate being looked after Clients who don't want to be independent
	Family and/or caretakers' behaviour and attitude		Family and caretakers are more empowered and outspoken Family and caretakers have the feeling client have the right to receive care Family and caretakers have distorted view of care needed by clients Family and caretakers having high demands and expectations: for example want to receive specific care
	Clients' motivation	It is motivating for clients to take the time to guide them to self‐care. At first it takes time, but afterwards it saves time, because the clients are able to take care of (partly) themselves It is motivating to show clients the benefits	Clients trying out multiple alternatives without success works demotivating Inconsistency of performing interventions by team can create confusion for clients Clients that are not willing and motivated to increase their self‐care
	Clients, family and/or caretakers' knowledge	Availability of educational materials for client and caregivers	Clients, family and caretakers not being aware of the urgency to change
3	Behaviour and attitude of the other individual healthcare professional^c^	Use of support services such as social work or community teams Being up‐to‐date of care innovations/technology/aids	Raising expectations of clients and/or their carers towards the care and the volume of care by other healthcare professionals. Prescribing unnecessary frequency of interventions Prescribing care that's not in line with (nursing) guidelines Prescribing unsuitable materials for the prescribed care Other healthcare professionals deciding and prescribing care that is not within their authority and responsibility. Not stimulating self‐care of clients in the hospital Differing or conflicting views on care
	Behaviour and attitude within homecare teams	Receive regular, brief and concise updates form guidelines specified within their homecare team. Discuss necessary care in consultation with general practitioners Involving other disciplines to tackle low‐value care Client meetings in the team	Conflicting vision or attitudes of team members towards what constitutes ‘good care’ Team culture Agreements are not followed by the entire team Frequency and lack of meetings to communicate agreements Engaging with other disciplines is seen as difficult experience
	Interactive behaviour and attitude of other healthcare professionals towards homecare professionals^c^	Regular interdisciplinary interaction facilitates interdisciplinary communication Tackling low‐value care practices together, for example working together with occupational therapist to empower client self‐care Returning incorrect care requests to the requesting healthcare professional	Interdisciplinary communication is seen as difficult Receiving incorrect care requests from other healthcare professionals Image of profession and their tasks
	Cooperation between hospitals and homecare organizations	Start practicing in hospital to work towards self‐care Screening care requests from the hospital by a care intermediary before entering care	Transfer care from hospital to homecare proceed not as agreed Returning incorrect care requests to the requesting hospital
4	Attitude of clients towards incentives and resources	Take the time to guide clients to encourage self‐care	Not willing to pay for aids or out‐of‐pocket payments
	Attitude of homecare professionals towards incentives and resources	Professional autonomy	Yielding to low‐value care results in less work
	Role of health insurers in incentives and resources		Non‐reimbursement of meeting or discussion time for homecare professionals Non‐reimbursement of aid offers care aids/materials for clients
	Role of homecare organizations in incentives and resources	Opportunities to practise with different care aids before the client buys them Organization could facilitate materials and care aids Shadowing colleagues Manage expectations: explicit agreements on what the organization offers and doesn't offer Step‐by‐step plan for taking on clients	Shortage of homecare professionals Financial incentives of the organization for delivering low‐value care Competition between organizations results in taking on care requests
5	Mandate/authority/accountability of homecare professionals	Capable leadership	Lack of authority or having a say in decision making Experience a top‐down management
	Mandate/authority/accountability of the organization	A plan to inform the entire organization on innovations or (de‐) implementations Multi organizational cooperation to address regional projects and problems	Teams are not adequate included or informed about new (de)‐implementations
6	Funding of home‐based nursing care		Production stimulus (price x quantity) Experience lack of space to request (extra) time Care aids and supplies are not (sufficiently) reimbursed Experience no funding for support services, such as volunteers or social workers
	Homecare organization	Stimulate projects to promote self‐care	Lack of cooperation and fragmentation of home‐based nursing care organizations
	Societal awareness	Media attention to increase the sense of urgency to de‐implement low‐value care urgency	
	Influential people	Person of contact in de organization as role model Organizational support for the de‐implementation	

*Note*: Domains 4, 5 and 6 will be described in ‘external factors’.

^a^ TICD domains: (1) individual health professional factors, (2) client and relatives' factors, (3) professional interactions factors, (4) incentives and resources factors, (5) capacity for organizational change factors and (6) social, political and legal factors Flottorp et al., 2013.

^b^ Relevant Understandable, Measurable, Behavioural and Attainable (RUMBA).

^c^ Other healthcare professionals, for example general practitioners/medical specialists and occupational therapist.

#### 5.2.1 Barriers of low‐value home‐based nursing care

##### The individual health professionals' factors

As illustrated in the following quote, homecare professionals expressed their willingness to change current practices, but at times, they do not feel the urgency to discontinue low‐value care practices, for example because of social monitoring of clients. Homecare professionals also considered daily routines as a reason for providing low‐value care. According to them, if new practices are not regularly repeated before becoming embedded, there is a risk of reverting to old habits.

*It's not so much willingness, it's sometimes necessity and that also has to do with the sense of urgency, some don't see that*. *Focus group interview 6*

Homecare professionals also sometimes feel the need to offer the client ‘something’, because it goes against their nature to step back and offer less. On the other hand, homecare professionals also mentioned engaging in low‐value care practices because it would take less time than if the client were to do it themselves. Additionally, they might do so to avoid difficult conversations with resistant relatives or agitated clients, as the following quote illustrates. Moreover, homecare professionals might experience pressure from clients, for example when clients claim that a colleague still provides the care that the homecare professional refused to do.

*Sometimes I think that if you don't go along with it, then you have a lot more work than if you do go along with it, and maybe you will deliver low‐value care, but in the end, it's less work than dealing with angry families or agitated clients*. *Focus group interview 1*

In addition, homecare professionals often experience a lack of summary and specific details within guidelines. For example, there might be a lack of information on the frequency of performing an intervention on a daily basis. Furthermore, homecare professionals also find that guideline recommendations in general are not always directly applicable in the context of homecare. With regard to compliance with guidelines, homecare professionals feel they have less control within the homecare context and are more dependent on the specific situations they encounter compared to intramural care settings, as the following quote illustrates.

*Guidelines are often not concrete enough such that you can literally cut and paste them in a situation. In the homecare situation we are dependent on all sorts of factors. When you have a guideline in an intramural setting, you have more influence on factors. We have less influence on that, and we just have to deal with what we got*. *Focus group Interview 3*

Homecare professionals also acknowledged that while they themselves may lack specific skills, they also note a lack of professional skills among colleagues in their team. An example of this is the ability to engage in conversations with other healthcare professionals or clients and relatives. They also note a lack of skills for the use of care aids, as well as digital skills in their team. Furthermore, another skill for homecare professionals (level 6) which needs improvement is ‘conduct of needs assessment’ as variance in this procedure is observed between teams in one organization.

##### Professional interactions' factors

Homecare professionals believed that team culture also influenced the provision of low‐value care. Achieving consensus on the quality of care is mentioned as challenging because each team member had their own view on care quality, as shown in the following quote. In addition, homecare professionals also encountered difficulties in ensuring that team members adhered to their agreements. The limited opportunities for communication in homecare may further complicate reaching a mutual understanding within the team.

*You can never get everyone exactly on the same page. Everyone will always have a personal view I think*. *Focus group interview 4*

In addition, the homecare professionals also observed barriers in the collaboration with other healthcare professionals external to the homecare organization – such as general practitioners or hospital staff—who are often unaware of the work responsibilities of a homecare professional. Furthermore, the transition from hospital to homecare often does not proceed as initially agreed on. Homecare professionals also experienced that clients’ self‐care is not sufficiently addressed in hospitals, as the following quote demonstrates:

*We very often notice that nothing is done with self‐care in hospitals. I have worked in hospitals myself. It is sometimes more efficient for a nurse to do it quickly […] but there is no working towards self‐care, not at all. Actually I think a lot of care could be prevented if this was already being worked on in the ward*. *Focus group interview 6*

Similarly, creating expectations among clients and their support networks and the prescription of low‐value care by other external homecare professionals promoted the use of low‐value care.

##### Client and relatives' factors

From the perspective of homecare professionals, some clients and their networks are more outspoken about the care they believe they are entitled to. Homecare professionals have also encountered high and unrealistic expectations from clients about the care they should provide, as evident in the following quote:

*In addition, I think there is another expectation from clients about what we can offer. When clients call to request care, they often expect us to be at their doorstep every day at 8 a.m. and make them a sandwich. And if we do the shopping, they are completely happy, but that is totally unrealistic given the current situation. I see this with a lot of new clients, but with old clients [client who receive care for a longer period] too*. *Focus group interview 7*

Homecare professionals expect that clients who have received care for an extended period may pose a challenge in that they may be very hard to motivate for agreeing with reducing low‐value care, as demonstrated in the following quote:

*Yes, I also think that it is mostly the category of clients that received care for a longer period of time. I don't know if you can change the habit of receiving care and I don't know what it takes to change it*. *Focus group interview 1*

Homecare professionals have also previously encountered situations where these ‘long‐term clients’ switched to other organizations when their care was reduced, as the following quote demonstrates:

*[the client] had received care from our organization for 12 years, but [the client] didn't agree to reduce the care. We were planning to reduce the care and the client sought another organization that would be willing to provide the care we reduced*. *Focus group interview 2*

##### External factors

Homecare professionals held the belief that the role of health insurance in reducing low‐value care was primarily associated with the non‐reimbursement of materials or care aids, such as a care aid for putting on or changing compression stockings. These expenses are often incurred by clients who are either unable or unwilling to cover the costs themselves. Healthcare organizations typically hesitate to cover these costs for clients. Homecare professionals have also noted the need of time to educate clients on the use of care aids, yet they face constraints in requesting extra time:

*I think that you temporarily need more time to calmly guide and encourage the client. We don't get that, that time, which is why everything gets squeezed in and then it's hurry, hurry and then you don't convince the client*. *Focus group interview 3*

On the other hand, homecare professionals also mentioned that financial incentives within the organization of care played a role. Currently, there is a strong emphasis on dedicating more time to clients rather than less, as illustrated in de following quote:

*You have to make your hours with those clients anyway, because those are the productive [claimable] hours*. *Focus group interview 5*

Homecare professionals suggested that organizations were in competition with each other and that it could be helpful to counter this competition with collaboration between organizations to jointly address projects and issues, as demonstrated in the following quote:

*We also suffer from market forces in healthcare. Because one organization does something and the other can't. We all work independently […] we all pay [the three healthcare organizations in the city name] the same price for travelling and consulting. Still, we compete with each other and we all just stand in the same apartment block looking at the same doors*. *Focus group interview 5*

#### 5.2.2 Facilitators of low‐value home‐based nursing care

##### The individual health professionals' factors

Healthcare professionals indicate that reflecting on current practices encourages reducing low‐value care. It could create awareness, ensure that old patterns are broken, and embed new innovations. Reflecting together with their team could facilitate their transformation, as illustrated in the quote below:

*Why do you do the things you do? Do you do them because others do them, do you do them for the sake of doing them or could you do them in a different way? Just discuss this […] with your colleagues*. *Focus group interview 1*

Homecare professionals emphasized that staying updated on new technological innovations and care aids is essential for reducing low‐value care. They would also appreciate receiving notifications about adjustments to guideline recommendations in general. In this way, they are immediately informed about new developed guidelines and they also prefer that adjustments are marked in the guideline, as described in the following quote.

*When guidelines have been amended, you should be informed about which guidelines have been amended and that they mark for example in red font in the guidelines such that the changes immediately stand out*. *Focus group interview 2*

To address the lack of knowledge and skills, they suggested that taking courses or receiving training to develop such skills would be helpful, for example guidance on how to engage conversation reducing this care. In the quote below, a homecare professional took the initiative by assisting colleagues in developing their skills to use a care aid for compression stockings.

*For example, with the new care aid [brand name], some colleagues did not know how to use the care aid and they had to find out at the client's home. […] We put a [brand name] to practice at the office. When you provide enough space and care aids to get experienced, then it is okay*. *Focus group interview 2*

To improve conducting needs assessments and counteract variation between team within the organization, healthcare professionals (level 6) suggested that it would be helpful to get guidance on conducting needs assessments and to have a clear policy on this matter, as the following quote illustrates:

*Homecare organizations should make a clear policy with the people who conduct needs assessments. We all conduct need assessments differently, so some would request time to provide guidance and others would not, because they don't see the need for it or the advantages to provide guidance to clients*. *Focus group interview 3*

Homecare professionals (level 6) commented that when agreements, goals and boundaries are established and communicated from the start of providing care, clients are generally more willing to agree to these agreements. When establishing and communicating expectations with clients and their support networks, it is important to consider the clients’ level of self‐care and the involvement of their network.

##### Professional interactions' factors

To improve the communication within teams, one team held weekly meetings in which a team member serving as a contact person for several clients discussed these clients’ progress and assistance requests. This approach allowed for maintaining an overview of the clients, the establishment of agreements and the identification of activities to be addressed in a subsequent meeting, as exemplified in the following quote:

*In the team, I am the only homecare nurse [level 6] and the rest of the colleagues are all contact persons for clients, but I have to keep an overview and if everyone is doing their thing then I have no idea what still needs to be done and how things are going. So I thought, well, let's just meet once a week with the colleagues who are working and discuss their clients […] Then I just keep a bit of an overview of the clients*. *Focus group interview 1*

To enhance collaboration and interaction with other healthcare professionals within and outside the organization, homecare professionals suggested involving other disciplines in approaches to reduce low‐value care and discussing together what care is required for the client. For example, having an occupational therapist assesses what clients could do themselves.

##### Client and relatives' factors

In contrast to the barriers for client and relatives, some clients prefer to be independent and are more motivated to reduce care and regain their independence. During the COVID‐19 pandemic, homecare professionals noticed an increase in self‐care among many clients, as they preferred to limit the number of visitors in their homes, as the following quote illustrates. This goes to show that when clients were motivated, there are opportunities to reduce low‐value care.

*In the first wave of COVID, it amazed me how many people who received care could do things independently. From I am too sick, too weak, too nauseous. From I can't do it by myself to we're going to do it ourselves. You can come only twice a week*. *Focus group interview 1*

##### External factors

Regarding the barriers ‘lack of time for educating clients’ and ‘additional costs of unused care aid’, homecare professionals suggested that facilitating clients by homecare organizations in practising with and finding the appropriate care aid before purchasing it could be helpful in the use of the care aid and clients' willingness to pay, as demonstrated in the following quote:

*[…] If you have the right care aid, the motivation for using it, and you take the time to teach the client how to use it, then it's easier […]. I also think that some people end up not being able to use it themselves, so if you have bought an expensive care aid like [brand name] or [brand name] […] clients end up not being able to use it themselves, you […] spend a lot of money on something that you, as a client, can no longer use*. *Focus group interview 2*

Homecare professionals also pointed out that community and healthcare organizations are promoting projects aimed at fostering clients’ self‐care. For example, one project focuses on enabling older persons to live longer at home while addressing issues like loneliness and self‐care. Activities are organized to help them connect with peers. Lastly, it was emphasized that having support for de‐implementation from the organization, including from managers, was considered important, as illustrated in the following quote:

*I think it is also good that you have the support of the organization. So, when there is trouble, a client is acting difficult, or doesn't want to cooperate with new developments, that an organization or manager support you and may also be prepared to do something about it*. *Focus group interview 2*

## 6 DISCUSSION

This study aimed to gain insight into barriers and facilitators perceived by homecare professionals regarding the reduction of low‐value home‐based nursing care. Barriers and facilitators for individual homecare professional included factors such as awareness, behaviour, attitude and lack of skills, aligning with the findings of van Achterberg et al. (2008) and Halm (2022). According to Halm (2022), the success of de‐implementation is more likely when healthcare professionals are aware of both human and system barriers and facilitators.

A specific barrier for reducing low‐value home‐based nursing care within the homecare environment mentioned was the ‘variation in conducting needs assessments’. This variation implies that clients with the same medical condition may receive different care without a specific explanation. In the homecare environment, person‐centred care could contribute to the variation in needs assessment, because client preferences are included (Brabers et al., 2019). In needs assessments, homecare nurses collaborate with the client and the client's support network in making decisions regarding the care needed, with the goal of enhancing the client's self‐care and self‐management skills. Schwenke, van Dorst, et al. (2023) also pointed out that homecare nurses could benefit from more guidance in conducting needs assessments. This issue corresponds to the facilitator ‘receiving guidance in conducting needs assessments’ mentioned by the homecare nurses in our study, emphasizing the need for guidance, a clear policy for needs assessments and reducing low‐value care.

Homecare nurses and nursing assistants are primarily playing an executive role and may perform these low‐value care practices when they are requested by general practitioners. The homecare professionals in our study experienced requesting low‐value home‐based nursing care by general practitioners as a barrier. There are several drivers why general practitioners (in the Netherlands) prescribe low‐value care, including wanting to maintain a good relationship with clients and wanting to offer the client something, lack of time and lack of knowledge (Kool et al., 2020). In a study of Augustsson et al. (2021), expectations, attitudes and behaviours of physicians were also related to higher use of low‐value care. In our study, homecare professionals indicated that discussing low‐value care and involving other disciplines would facilitate addressing these low‐value care practices. A specific example in the homecare environment is the early involvement of occupational therapists. In the context of the ‘reablement’ project in the Netherlands, different disciplines work together in an integrated approach to establish shared goals by clients, their relatives and healthcare professionals (Clotworthy et al., 2021). Working in interdisciplinary teams was experienced as positive, strengthening team members' professional identities, broadening their professional competencies and fostering a sense of community and mutual support (Birkeland et al., 2017; Hjelle et al., 2018).

From the perspective of homecare professionals, barriers related to clients and relatives encompass attitudes, behaviours and a lack of knowledge. Examples of attitude and behaviour barriers in our study include clients and relatives feeling entitled to receiving care, the habit of expecting care for extended periods, clients and relatives being assertive about the care the client should receive and clients and relatives having high and unrealistic expectations of care. The latter finding aligns with the finding of Augustsson et al. (2021), where patients showed high expectations and a lack of knowledge, leading them to request low‐value care practices from physicians. Patients interviewed in the studies by Verkerk et al. (2023) and Sypes et al. (2020) suggested that if physicians had sufficient time and the necessary educational materials, they could better inform them about different care options and their benefits and drawbacks, thereby promoting more realistic expectations of care. In our study, the use of educational materials for clients and motivating them by showing the benefits of self‐care were seen as facilitators to ensure that clients become motivated to reduce low‐value home‐based nursing care. We anticipate that enhancing communication between nurses, nursing assistants and clients—by engaging clients, using educational materials, demonstrating benefits, communicating expectations and taking the time to inform clients—could facilitate the reduction of low‐value home‐based nursing care.

External factors identified as barriers include non‐reimbursement of materials for clients and resources for homecare professionals and financial incentives for organizations to promote the provision of low‐value care. Stimulation of financial incentives from organizations was also observed among physicians, as some payment models prioritize the volume of care over reducing care (van Dulmen et al., 2020). In the Dutch homecare system, homecare nurses and nursing assistants are compensated for the hours they spend providing care. This policy can create financial incentives to provide more care instead of reducing it. According to homecare professionals in the present study, organizational support for de‐implementing low‐value care could facilitate the reduction. However, it is important to note that this support does not guarantee that a healthcare organization will save costs when de‐implementing low‐value care, as outlined by Kroon et al. (2023). This challenge extends to government and health insurance companies, according to Kroon et al. (2023). Still, these financial considerations should not deter the efforts to de‐implement low‐value care. From a societal point of view, de‐implementing of low‐value care is seen as providing care of higher quality, promoting efficient use of time and resources in healthcare and addressing the shortage of homecare professionals (Kroon et al., 2023). This shortage is a barrier, currently also experienced by homecare professionals in their efforts to reduce low‐value home‐based nursing care, because they need time to guide clients towards self‐care.

The findings presented in this paper can assist in the development of de‐implementation strategies aimed at reducing low‐value home‐based nursing care. Implementation mapping, an approach derived from intervention mapping, can aid in developing de‐implementation strategies by aligning identified barriers and facilitators with the specific context of de‐implementation (Fernandez et al., 2019; Kok et al., 2017). In our study, we identified several barriers to de‐implementation, such as the ‘lack of knowledge about low‐value care’, ‘reluctance of clients and relatives in reducing low‐value care’ and ‘lack of cross‐professional collaboration’. To address these, potential strategies include ‘conducting educational meetings’, ‘involving clients and relatives in the process of reducing’ and ‘promoting network weaving’ (Powell et al., 2015). Effectively de‐implementing low‐value home‐based nursing care requires a structured plan incorporating multiple strategies across all involved stakeholders (Augustsson et al., 2021; van Bodegom‐Vos et al., 2017; van Dulmen et al., 2020). In the context of implementation mapping, a step‐by‐step plan is formulated, encompassing the development, de‐implementation and evaluation of strategies within an iterative process (Fernandez et al., 2019; Kok et al., 2017).

### 6.1 Strengths and limitations

One of the strengths of our study is that it is the first to examine barriers and facilitators of reducing low‐value home‐based nursing care. These insights enable the development of de‐implementation strategies and create opportunities for more appropriate care. Another strength is that it presents the perspective of homecare professionals who are directly involved in low‐value home‐based nursing care practices. They possess valuable insights into the different factors driving these practices and can identify the key stakeholders involved. In addition, it is a strength that 27 teams from different organizations from a wide region in the Netherlands have been reached, with different levels of education and homecare experience. Furthermore, the data were analysed with a broad group of experts, increasing the trustworthiness of the findings. On the other hand, the interviews were performed by different researchers due to logistical and geographical reasons. Although they used the same interview guide, it is possible that this had led to observer bias. In addition, while homecare professionals provided insights about other stakeholders, including general practitioners, occupational therapists, clients and clients' relatives, any real experienced barriers and facilitators experienced by these stakeholders were not captured. This information would be relevant to develop tailored de‐implementation strategies for these stakeholders.

### 6.2 Recommendations for further research

Further research is needed on the effectiveness of tailored de‐implementation strategies and the overall process of de‐implementation. In addition, further examining barriers and facilitators experienced in collaboration with other disciplines, such as general practitioners or hospital staff, and developing de‐implementation strategies focused on these stakeholders as well are recommended.

## 7 CONCLUSION

We explored specific barriers and facilitators faced by homecare professionals in reducing low‐value home‐based nursing care across multiple homecare organizations. Professionals identified lack of knowledge and skills, for example to use care aids, and variation in needs assessments, as a barrier. Providing guidance and training could enhance development of these skills, ensuring adherence to guidelines and team agreements. Additionally, expectations from clients and relatives, as well as prescriptions for low‐value care by other healthcare professionals, were noted as barriers. Promoting cross‐professional and cross‐organizational collaboration could facilitate tackling these practices together. Involvement of relatives and encouraging clients to self‐care could help addressing these issues. Externally, non‐reimbursement of care aids, additional cost for clients and not using the purchased care aids encouraged the provision of low‐value care. Allowing clients to practise with care aids before purchase could facilitate their use and eventually replace the need for homecare professionals to provide this care. These insights form the foundation for developing tailored de‐implementation strategies to reduce low‐value home‐based nursing care, creating opportunities for more appropriate care and accommodating waitlisted clients.

## AUTHOR CONTRIBUTIONS

Milou Cremers: Equal first authors, conceptualization, methodology, investigation, data collection, data curation, data analysis, writing—original draft, writing—review & editing, supervision, project administration. Benjamin Wendt: Equal first authors, conceptualization, methodology, investigation, data collection, data curation, data analysis, writing—original draft, writing—review & editing, supervision, project administration. Getty Huisman‐de Waal: Conceptualization, methodology, data collection, data analysis, writing—review & editing, supervision, project administration. Leti van Bodegom‐Vos: Conceptualizing, writing—review& editing, final review. Simone van Dulmen: Conceptualizing, data collection, writing—review & editing, final review. Elise Schipper: Conceptualizing, data collection, data analysis, writing—review & editing, final review. Monique van Dijk: Conceptualizing, writing—review& editing, final review. Erwin Ista: Conceptualization, methodology, data analysis, writing—review & editing, supervision, project administration.

## FUNDING INFORMATION

This study was funded by ZonMw, the Netherlands Organisation for Health Research and Development (grant numbers 10110032010003 and 10110032010002).

## PEER REVIEW

The peer review history for this article is available at https://www.webofscience.com/api/gateway/wos/peer‐review/10.1111/jan.16381.

## Supporting information


Appendix S1.

Appendix S2.

Appendix S3.

## Data Availability

All participants of the interviews signed an informed consent giving permission to use the data. Data is available on request from the authors.
