# Supplementary material for: Barriers and facilitators for reducing low‐value home‐based nursing care: A qualitative exploratory study among homecare professionals
Source: J Adv Nurs. 2024 Aug 22;81(11):7167–80. doi: 10.1111/jan.16381 (PMC12535367; doi:10.1111/jan.16381)
Supplement: Supplementary file 1 — Appendix S1. [file JAN-81-7167-s003.docx]

**Appendix A: Interview guide**

- Introduction of the topic

“The topic of the focus group interview is low-value home-based nursing care practices. These are practices of low value or no value to the clients. We would like to discuss reasons for providing these practices. We would like to emphasize that performing these practices is neither right nor wrong, and can also depend on the client’s wish. That is why we would like to elaborate on these reasons. We also want to explore whether we can reduce these practices in certain situations. We want to look at barriers to reduce these practices and facilitators that can encourage reducing low-value home-based nursing care practices.”

Start data collection:

- Opening question:
  - To what extent do you provided these practices and what is your reaction to the idea that these practices are considered as low value in home-based nursing care (LVHBNC).
- Questions for barriers
  - What factors cause that these [LVHBNC] are provided?
  - What factors complicate reducing these [LVHBNC]?
- Questions to continue the conversation based on the Tailored Implementation for Chronic Diseases checklist (TICD) (Flottorp et al., 2013).
  - What do you think about the fact that, for example, [LVHBNC] is not recommended according to the latest guidelines?
  - What do you think about available literature/guidelines and protocols on these LVHBNC? Consider clarity, availability, accessibility, quality and evidence, strength of recommendation, sources and consistency of guidelines.
  - What information do you need or want to know for stopping or reducing the LVHBNC
  - What might help you become aware or more conscious of your own actions?
  - To what extent do you think the recommendations will lead to desired outcomes? For example, time savings, improved client outcomes or fewer wasted materials?
  - What barriers do you expect from your team to stop/reduce the LVHBNC?
  - What reactions do you expect from the clients who were previously receiving the LVHBNC? Would they be open to the change?
  - What reactions are expected from other healthcare professionals (e.g., general practitioner, (nursing) specialist)
  - In what ways do safety and quality improvement staff members influence performing LVHBNC?
  - What organizational aspects hinder changes?
  - What influence does the health insurer have on the barriers?
  - Might certain individuals in certain positions hinder changes? Do not mention names here, but what barriers would be involved?
  - How would budget, liability, legislation or agreements affect changes?

----------------------------------------------------**Summary of the discussion**----------------------------------------------

- Questions regarding facilitators
  - What would help you to stop or reduce LVHBNC?
  - Have you ever stopped LVHBNC before?
- Questions to continue the conversation based on the TICD.
  - What was your experience with that?
  - What was the success factor in reducing?
- Questions to continue the conversation based on the TICD.
  - What would motivate you to change your own actions?
  - What would motivate you or your colleagues to stop or reduce LVHBNC?
  - What motivating influence can clients have in reducing a particular LVHBNC?
  - What is your team's influence on stopping/reducing LVHBNC?
  - What materials/resources are needed to encourage a change?
  - What support do you need/has the team needed? For example: decision aids, supervision, client information
  - What organizational aspects are needed to encourage, motivate and support the change?
  - Who are the key people that could stimulate/support the change?
  - What external support is needed to support the changes?
  - What is the impact of budget, liability, legislation or agreements?

----------------------------------------------------**Summary of the discussion**----------------------------------------------

- Completion
  - Anything else you guys want to say on this topic? Did I forget to ask anything?
  - What did you find most important in this session / What will you remember?
  - How did you experience the session?
  - Reminder for the member check
  - Thank them for active participation
  - Take notes or leave recorder on until participants have left

---------------------------------------------------------**Turn** **of the recorder**--------------------------------------------------

- Evaluation (moderator and assistant)
  - Review of impressions, themes and notes
  - If possible, compare with previous focus group interviews
  - Evaluate roles and performances of moderator and assistant
  - Evaluate the quality of the focus group (e.g., responses, interactions and behavior)
  - Evaluate time schedule
  - Discuss notes and first impression of findings.

Flottorp, S. A., Oxman, A. D., Krause, J., Musila, N. R., Wensing, M., Godycki-Cwirko, M., Baker, R., & Eccles, M. P. (2013). A checklist for identifying determinants of practice: a systematic review and synthesis of frameworks and taxonomies of factors that prevent or enable improvements in healthcare professional practice. *Implement Sci*, *8*, 35.
