# Supplementary material for: Barriers and facilitators for reducing low‐value home‐based nursing care: A qualitative exploratory study among homecare professionals
Source: J Adv Nurs. 2024 Aug 22;81(11):7167–80. doi: 10.1111/jan.16381 (PMC12535367; doi:10.1111/jan.16381)
Supplement: Supplementary file 2 — Appendix S2. [file JAN-81-7167-s001.docx]

**Appendix B: educational and professional status**

| Educational, professional status and job descriptions of homecare professionals in the Netherlands | | |
| --- | --- | --- |
| **Profession** | **Educational level*** | **General task description** |
| Nurse Practitioner (*Master’s degree)* | Level 7 | Practitioner with both nursing and medical expertise – diagnosing patients – needs assessment and coordination of care and medical treatment – responsible for quality of care and team expertise |
| Registered Nurse (*Bachelor’s degree)* | Level 6 ** | High complex nursing and care – responsible for quality of care and team expertise – coaching colleagues – coordination of care – needs assessment |
| Registered Nurse  *(Vocationally trained)* | Level 4 | (Complex) nursing and care – coordination on patient level |
| Certified Nursing assistant | Level 3 | Low complex nursing, care and support – care plan |
| Health and Welfare assistant | Level 2 | Domestic and light care tasks (daily activities) |
| Note:  * According to Dutch Qualification Framework (NCP NLQF, 2019)  ** Registered nurses have the responsibility of conducting needs assessments with the client and the client’s network to determine the necessary nursing care in light of strengthening the client’s self-reliance and self-management (Schwenke et al., 2023). | | |

**References**

NCP NLQF. (2019). *Dutch Qualification Framework (NLQF): Increases visibility and value of learning*. <https://www.nlqf.nl/images/downloads/Artikelen/NLQF_Brochure_Engels_2018_site.pdf>

Schwenke, M., van Dorst, J., Zwakhalen, S., de Jong, J. D., Brabers, A. E. M., & Bleijenberg, N. (2023). Measures to improve patient needs assessments and reduce practice variation in Dutch home care organizations. *Nurs Open*, *10*(5), 3052-3063.
