# Supplementary material for: Barriers and facilitators for reducing low‐value home‐based nursing care: A qualitative exploratory study among homecare professionals
Source: J Adv Nurs. 2024 Aug 22;81(11):7167–80. doi: 10.1111/jan.16381 (PMC12535367; doi:10.1111/jan.16381)
Supplement: Supplementary file 3 — Appendix S3. [file JAN-81-7167-s002.docx]

**Appendix C: The list of low value home-based nursing care practices presented during focus group interviews**

| Low value home-based nursing care practices *: |
| --- |
| - Washing the client from head to toe daily |
| - Washing with water and soap by default |
| - Bladder irrigation to prevent clogging of urinary tract catheter |
| - Bladder irrigation to prevent urinary tract infection |
| - Assist with (un)dressing while the client ca do this him/herself |
| - Assist with washing while the client ca do this him/herself |
| - Re-use of a urinary catheter bag after removal/disconnection |
| - Use an extra inlay to prevent leaking of continence material |
| - Assist with putting on/taking off compression stockings while the client can do this him/herself (possibly with an care aid) |
| - Daily application/removal for the night or replacement of bandages without a specific reason |
| - Choosing short-stretch bandages by default instead of using techniques such as Coban, UrgoK2, FarrowWrap of JuxtaLife |
| - Daily changing of a urinary catheter |
| - Application of zinc cream, powders or pastes when treating intertrigo |
| - Measuring vital signs (blood pressure, temperature, pulse, respiration rate) without a specific reason |
| * These low value home-based nursing care practices is presented in a random order |
